# LncRNA PCAT6 promotes the occurrence of laryngeal squamous cell carcinoma via modulation of the miR-4731-5p/NOTCH3 axis

**DOI:** 10.1093/jrr/rrae042

**Published:** 2024-07-02

**Authors:** Jianye Bai, Fen Yao, Yeyun Fu, Ningxin Kang, Guohua Wen

**Affiliations:** Department of Otorhinolaryngology, Yancheng First People's Hospital, Yancheng Clinical College, Jiangsu University, Yancheng, 224000, Jiangsu, China; Department of Anesthesiology, Yancheng First People's Hospital, Yancheng Clinical College, Jiangsu University, Yancheng, 224000, Jiangsu, China; Department of Otorhinolaryngology, Yancheng First People's Hospital, Yancheng Clinical College, Jiangsu University, Yancheng, 224000, Jiangsu, China; Department of Otorhinolaryngology, Yancheng First People's Hospital, Yancheng Clinical College, Jiangsu University, Yancheng, 224000, Jiangsu, China; Department of Otorhinolaryngology, Yancheng First People's Hospital, Yancheng Clinical College, Jiangsu University, Yancheng, 224000, Jiangsu, China

**Keywords:** laryngeal squamous cell carcinoma, PCAT6, miR-4731-5p, NOTCH3, malignant tumor

## Abstract

Laryngeal squamous cell carcinoma (LSCC) is one of the most aggressive cancers that affect the head and neck region. Recent researches have confirmed that long non-coding RNAs (lncRNAs) present an emerging role in diversiform diseases including cancers. Prostate cancer-associated ncRNA transcript 6 (PCAT6) is an oncogene in lung cancer, cervical cancer, colon cancer and gastric cancer, but its role in LSCC is still unknown. In the current study, we attempted to figure out the role of PCAT6 in LSCC. RT-qPCR was to analyze PCAT6 expression in LSCC cells. Functional assays were to uncover the role of PCAT6 in LSCC. Mechanism assays were to explore the regulatory mechanism behind PCAT6 in LSCC. PCAT6 exhibited higher expression in LSCC cells and PCAT6 strengthened cell proliferation and inhibited cell apoptosis. Furthermore, lncRNA PCAT6 modulated notch receptor 3 expression and activated NOTCH signaling pathway via serving as a sponge for miR-4731-5p. Taken together, lncRNA PCAT6 was identified as an oncogene in LSCC, which revealed that PCAT6 might be used as potential therapeutic target for LSCC.

## INTRODUCTION

Laryngeal squamous cell carcinoma (LSCC) is the second most common malignant tumor of head and neck squamous cell carcinoma. The cure rate of LSCC patients at early stages reaches 80 to 90%, while that of LSCC patients at advanced stages is only 60% [1]. Despite advancements in the treatment of LSCC, the overall survival rate of LSCC patients remains unfortunately low [2]. Therefore, it is urgent to identify effective biomarkers and explore novel therapeutic methods for LSCC.

Long noncoding RNAs (lncRNAs), referring to a type of non-coding RNAs, have been studied extensively in various cancers [3]. Accumulating evidence points out that lncRNAs act as regulators of most cellular processes to participate in human diseases and cancers [4]. For example, Wu *et al.* have revealed that lncRNA FOXD1-AS1 influences chemoresistance in gastric cancer by activating the PI3K/AKT/mTOR pathway [5]. Zhang *et al.* have clarified that lncRNA SNHG6 functions as a tumor promoter in glioma via miR-543/LMO3 axis [6]. Wang *et al.* have exposed that LINC01121 accelerates the development of breast cancer by sponging miR-150-5p and targeting HMGA2 [7]. Although the roles of lncRNA PCAT6 are studied extensively in various cancers, their role in LSCC is not well understood. In this study, we attempt to figure out the role of PCAT6 in LSCC and the latent regulatory mechanism in LSCC cells.

As reported, signaling pathways also exhibited great power on tumor progress. For example, NF-κB pathway exerts influence on the progress and tumorigenesis of tumors [8]. NOTCH pathway is involved in cell development and has abnormal expression in solid tumors [9]. The activation of Wnt signaling pathway also exert crucial roles in tumor development and growth [10].

Based on the role of PCAT6 in various tumors, we are interested in the expression levels of PCAT6 in LSCC and its potential functions and regulatory mechanisms. We aim to explore the role of PCAT6 in LSCC cells and its interactions with other molecules, signaling pathways or regulatory factors. By studying the mechanisms of PCAT6, we hope to determine whether PCAT6 can serve as a potential therapeutic target for LSCC.

## METHODS

### Cell culture and cell lines

All five cell lines were purchased for this study: four LSCC cell lines were AMC-HN-8, Tu 686, Tu 177 and FD-LSC-1 and one normal cell line NP69. Four cell lines (NP69, AMC-HN-8, Tu 177 and FD-LSC-1) were bought from Shanghai Honsun Biological Technology Co., Ltd (Shanghai, China). Tu 686 cell line was acquired from BioVector (Beijing, China). All cells have undergone STR identification and were cultured with 89% RPMI-1640 medium (Life Technologies/Invitrogen) supplemented with 10% FBS (GIBCO) and 1% penicillin/streptomycin (HyClone, Logan, UT), under condition of 37°C, 5% CO_2_.

### Plasmid transfection

LSCC cells with 80% density were inoculated into six-well plates, and Lipofectamine 3000 (Invitrogen, Carlsbad CA) was applied to carry out cell transfections consistent with producer’s protocol. Short hairpin RNA (shRNA) precisely targeted to PCAT6 was acquired from GenePharma (Shanghai, China). The pcDNA3.1 vector (Invitrogen) was bought to overexpress NOTCH3 for rescue assays. Besides, all miRNAs’ mimics, the miR-4731-5p inhibitor and negative control groups were purchased from GenePharma (Shanghai, China).

### Reverse transcription quantitative real-time polymerase chain reaction (RT-qPCR)

The usage of TRIzol Reagent (Invitrogen) was to extract the whole RNA from AMC-HN-8, Tu 686, Tu 177, FD-LSC-1 and NP69. The synthesis of cDNA was produced by using PrimeScript™ RT Master Mix (TaKaRa, Shiga, Japan), and SYBR green Supermix (Thermo Fisher, Waltham, MA) was applied to perform assays on Step-One Plus Real-Time PCR System (Applied Biosystems, Foster City, CA). miRNAs were detected by using Hairpin-it™ miRNAs qPCR Quantitation Kit (Genepharma, Shanghai, China). GAPDH and U6 were regarded as reference genes. Gene relative expression level was evaluated based on the 2^−ΔΔCt^ method.

### Cell counting kit-8 (C‌CK8)

A total of l × l0^4^ cells in each well were seeded into 96-well plates. Cell Counting Kit-8 (Dojindo Laboratories, Kumamoto, Japan) was used for detection of cell viability. After incubation for 24 h, 10 μl of CCK-8 solution (Dojindo, Japan) was added into plates with incubation at 37°C, 5% CO_2_ for 2 h. At last, cells were observed through a spectrophotometer (Thermo Fisher Scientific, Waltham, MA) with absorbance of 450 nm.

### 5-ethynyl-2'-deoxyuridine (EdU)

Cells were planted into 96-well plates overnight. After transfection with sh/NC, sh/PCAT6#1/2, sh/PCAT6#1 + miR-4731-5p inhibitor or sh/PCAT6#1 + pcDNA3.1/NOTCH3 for 48 h, the EdU kit (RiboBio, Guangzhou, China) and 4′,6-diamidino-2-phenylindole (Beyotime) were applied according to the manufacturer’s instructions. Cells were observed by fluorescence microscope (CKX41-F32FL, Olympus, Tokyo, Japan).

### 5,5',6,6'-tetrachloro1,1',3,3'-tetramethylbenzimidazolylcarbocyanine iodide (JC-1)

A total of 2 × l0^4^ cells were incubated into 12-well plates with 2.5 μg/ml of JC-1 dye (Beyotime, Shanghai, China) at 37°C for 30 min. The stained cells were washed with phosphate-buffered saline (PBS). The ratio of fluorescence cells was used for measuring the mitochondrial membrane potential.

### Western blot

RIPA lysis (Thermo) was purchased and used to extract protein samples, and the samples were separated by sodium dodecyl sulfate-polyacrylamide gel electrophoresis. Polyvinylidene fluoride membranes, to which proteins were transferred, were sealed with 5% skimmed milk at room temperature. Primary antibodies were then incubated with the membranes overnight at 4°C. These primary antibodies were bought from Abcam and the catalogues were: NOTCH3 (ab23426), Jag-1 (ab109536), Hes-1 (ab108937), Bax (ab32503), Bcl-2 (ab32124), Bcl-XL (ab32370) and GAPDH (ab8245). Secondary antibodies were incubated for 1 h in the dark. ECL luminous liquid (Pierce, Rockford, IL) was used to observe protein bands.

### Luciferase reporter assay

All pathway-related luciferase reporter plasmids are listed as follows: Myc luciferase reporter plasmid (Yeasen Biotech Co., Ltd, Shanghai, China), STAT3 luciferase reporter plasmid (Yeasen), TOP/FOP-Flash luciferase reporter vector (Millipore, Billerica, MA), NF-κB luciferase reporter plasmid (Yeasen), RBP-Jk reporter kit (SABiosciences, Frederic, MA), Oct4 luciferase reporter plasmid (Yeasen) and Nanog luciferase reporter plasmid (Yeasen). By inserting PCAT6 and NOTCH3 wide type or mutant fragments, which contain the binding sites of miR-4731-5p, into the luciferase reporter pmirGLO dual-luciferase plasmid (Promega, Madison, WI), luciferase reporter plasmids were established. After 48 h of transfection, luciferase activity was measured using a Luciferase Reporter Assay System (Promega).

### Subcellular fractionation

PARIS™ Kit (Ambion, Austin, TX) was employed to isolate cytoplasmic and nuclear RNA in line with manufacturer’s protocols. Cells were washed in PBS and then centrifuged at 500 × *g* for 3 min. Afterwards, cell samples were treated with cell fractionation buffer and disruption buffer. GAPDH served as the cytoplasmic reference, and U6 was seen as the nuclear reference. The isolated RNA levels in the nucleus and the cytoplasm were measured by RT-qPCR.

### RNA pull down assay

Biotinylated PCAT6 probe was used to treat cells and then incubated with magnetic beads. Magnetic beads were then added to the cells. The pull downs collected by beads were purified for PCR or western blot analysis. The experiment was independently conducted in triplicate.

###  RNA immunoprecipitation (RIP)

Based on the protocol of Magna RIP™ RNA-Binding Protein Immunoprecipitation Kit (Millipore), the RIP assay was conducted with the application of anti-Ago2 antibody or anti-IgG antibody. Cells were first collected and lysed in RIP lysis buffer containing protease and RNase inhibitors. The lysates were then conjugated with Ago2 antibody (Millipore) or negative control IgG (Millipore) on magnetic beads. Subsequently, the precipitated RNAs were collected and purified for RT-qPCR.

### Statistical analysis

Experiments were all repeated thrice at least, and results were calculated as the means ± standard deviations (S.D.). GraphPad Prism 5.0 software (GraphPad Software, Inc., La Jolla, CA) software was employed for statistical analysis with one-way analysis of variance (ANOVA) or Student’s *t* test. The significance level was identified statistically as the *P*-values below 0.05.

## RESULTS

### LncRNA PCAT6 is highly expressed in LSCC and promotes cell proliferation and restrains cell apoptosis

**Fig. 1:**
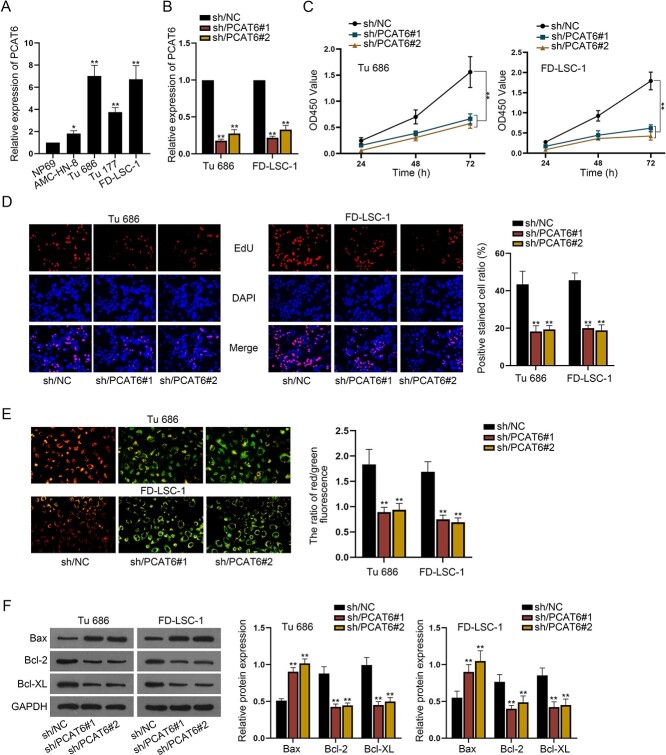
LncRNA PCAT6 is highly expressed in LSCC and promotes cell proliferation and restrains cell apoptosis. (**A**) RT-qPCR was to detect PCAT6 expression level in LSCC cells. (**B**) The interference efficiency of PCAT6 was assessed via RT-qPCR. (**C**, **D**) CCK8 and EdU assays were conducted to observe cell proliferation after PCAT6 was knocked down. (**E**) JC-1 assay was conducted to observe cell apoptosis. (**F**) Western blot was to analyze the protein levels of apoptosis-related factors in LSCC cells. ^*^P < 0.05, ^*^^*^P < 0.01.

To get a better understanding of the role of PCAT6 in LSCC, RT-qPCR analysis was to examine PCAT6 expression in LSCC cells. The results clearly showed that PCAT6 exhibited high expression in LSCC cells (Fig. 1A). Subsequently, we silenced PCAT6 in Tu 686 and FD-LSC-1 cells after transfection of sh/PCAT6#1/2 plasmids and detected the knockdown efficiency (Fig. 1B). From the experimental results of CCK8, it was overall observed that knockdown of PCAT6 greatly decreased the viability of Tu 686 and FD-LSC-1 cells (Fig. 1C). We also discovered that the positive-stained cells in EdU assays were remarkably reduced both in Tu 686 and FD-LSC-1 cells when PCAT6 was downregulated (Fig. 1D). Furthermore, cell apoptosis was examined by conducting JC-1 assays. The red/green ratio of negative group was even higher than that of sh/PCAT6#1/2 groups, indicating that PCAT6 depletion enhanced the apoptotic capacity of LSCC cells (Fig. 1E). Subsequently, western blot analysis was conducted to detect the protein levels of apoptosis-related factors (Bax, Bcl-2 and Bcl-XL) and the results demonstrated that after PCAT6 was silenced, the protein level of Bax was strengthened, while Bcl-2 and Bcl-XL protein levels were weakened (Fig. 1F). On the contrary, overexpression of PCAT6 in cells (AMC-HN-8) with relatively low expression levels promoted cell proliferation and inhibited apoptosis (Supplementary Fig. S1A–D). Collectively, PCAT6 was upregulated in LSCC cells and it functioned as a promoter in the occurrence of LSCC.

### PCAT6 modulates NOTCH3 expression and activates NOTCH pathway

**Fig. 2:**
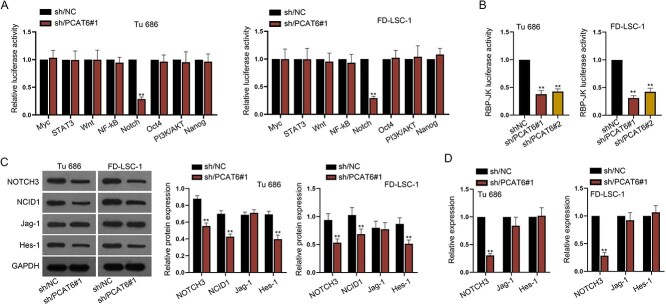
PCAT6 modulates NOTCH3 expression and activates NOTCH pathway. (**A**) Luciferase reporter assay was to examine the luciferase activity of signaling pathways when PCAT6 was downregulated. (**B**) RBP-JK luciferase reporter assay further confirmed that PCAT6 could activate NOTCH pathway. (**C**) Western blot assay tested the protein levels of NOTCH3, NCID1, Jag-1 and Hes-1. (**D**) RT-qPCR detected the mRNA levels of NOTCH3, NCID1, Jag-1 and Hes-1. ^*^^*^P < 0.01.

To explore the possible signaling pathways in the downstream of PCAT6, luciferase reporter assays were conducted. We observed that the luciferase activity of Notch signaling pathway was declined after PCAT6 knochdown, while no observable change was noted in the luciferase activity of other signaling pathways (Fig. 2A); conversely, overexpression of PCAT6 significantly increases Notch pathway activity, while having no significant effect on the activity of other signaling pathways (Supplementary Fig. S2A). Then, we implemented the RBP-JK luciferase reporter assay, confirming the association between PCAT6 and the NOTCH pathway (Fig. 2B, Supplementary Fig. S2B). Through western blot analysis, we discovered that the protein levels of NOTCH3, NICD1 and Hes-1 were notably decreased after transfection with the sh/PCAT6#1 plasmid (Fig. 2C). Besides, the mRNA level of NOTCH3 was found to be descended due to downregulation of PCAT6, suggesting that PCAT6 could activate NOTCH pathway by modulating NOTCH3 (Fig. 2D). In order to figure out the role of NOTCH3 in LSCC cells, we detected the expression of NOTCH3 in LSCC cells and it turned out that NOTCH3 had high expression in Tu 686 cells (Supplementary Fig. S3A). At the same time, rescue experiments were carried out to verify the interaction between PCAT6 and NOTCH3 in LSCC cells. CCK8 and EdU assays demonstrated that NOTCH3 overexpression enhanced the attenuated proliferative ability caused by PCAT6 deficiency (Supplementary Fig. S3B and C). The results of the JC-1 assay also showed that PCAT6 inhibition stimulated cell apoptosis, and this effect was reversed by NOTCH3 overexpression (Supplementary Fig. S3D). The similar result could also be seen in western blot analysis in Supplementary Fig. S3E. To sum up, PCAT upregulated NOTCH3 expression and thereby activated NOTCH pathway to influence LSCC cell proliferation and apoptosis.

### PCAT6 sponges miR-4731-5p to upregulate NOTCH3

**Fig. 3:**
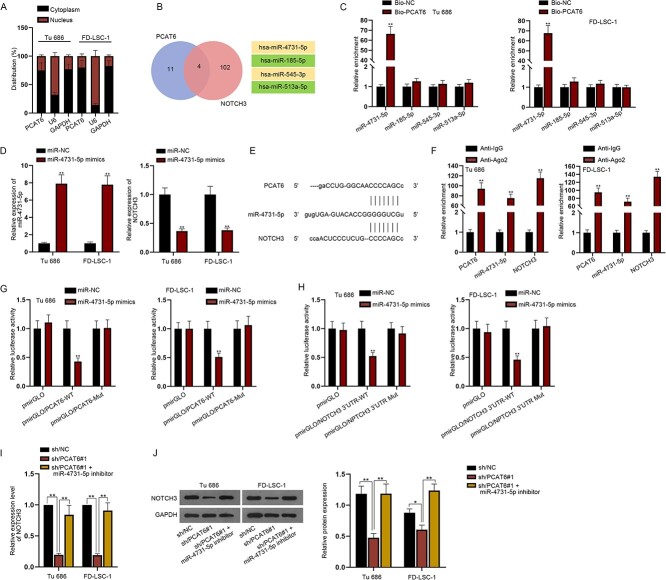
PCAT6 sponges miR-4731-5p to upregulate NOTCH3. (**A**) Subcellular fractionation was to explore the location of PCAT6 in LSCC cells. (**B**) starBase was utilized to screen out miRNAs that could bind to PCAT6 and NOTCH3. (**C**) RNA pull down assay displayed the enrichment of predicted miRNAs in the biotin-labeled PCAT6 probe. (**D**) RT-qPCR tested NOTCH3 expression after miR-4731-5p was overexpressed. (**E**) The potential binding sites between miR-4731-5p and PCAT6 or NOTCH3 were presented. (**F**) RIP assay was to confirm the relationship among PCAT6, mir-4731-5p and NOTCH3 in RISCs. (**G**, **H**) The interaction between miR-4731-5p and PCAT6 or NOTCH3 was verified via luciferase reporter assay. (**I** and **J**) RT-qPCR and western blot analysis were to detect NOTCH3 expression in sh/NC, sh/PCAT6#1 and sh/PCAT6#1 + miR-4731-5p inhibitor groups. ^*^P < 0.05. ^*^^*^P < 0.01.

Since we have known that PCAT6 could modulate NOTCH3 expression, mechanism assays were performed to probe the interaction between PCAT6 and NOTCH3. Hence, subcellular fractionation was carried out, and we found that the distribution of PCAT6 was mostly in the cytoplasm of LSCC cells (Fig. 3A), which suggests that PCAT6 might play its oncogenic role at the posttranscriptional level [11]. Thus, we made a conjecture that PCAT6 might be dependent on competing endogenous RNA (ceRNA) mechanism. CeRNAs are naturally occurring RNA molecules that compete with each other for binding to specific target. This competition can impact various cellular processes, including gene expression, RNA stability and protein function [12]. To confirm our hypothesis, we filtered four miRNAs (miR-4731-5p, miR-185-5p, miR-545-3p and miR-513a-5p) that could combine to both PCAT6 and NOTCH3 (Fig. 3B) by using starBase (http://starbase.sysu.edu.cn/) tool. Further, RNA pull down assay validated that only miR-4731-5p had a strong affinity with PCAT6 (Fig. 3C). Moreover, after transfection of miR-4731-5p mimics, we found that NOTCH3 expression was significantly lessened (Fig. 3D). The predicted binding sites between miR-4731-5p and PCAT6 or NOTCH3 are shown in Fig. 3E. Through Ago2-RIP assay, the co-existence of PCAT6, miR-4731-5P and NOTCH3 in RISCs was proved (Fig. 3F). Luciferase reporter assays were to confirm the interaction between miR-4731-5p and PCAT6 or NOTCH3 (Fig. 3G and H). Meanwhile, RT-qPCR and western blot analysis suggested that the reduced NOTCH3 expression on account of PCAT6 downregulation was completely restored by miR-4731-5p inhibitor (Fig. 3I and J). Briefly speaking, we identified that PCAT6 could act as a miRNA sponge to bind to miR-4731-5p and upregulate NOTCH3 in LSCC.

### PCAT6/miR-4731-5p axis promoting the growth ability of LSCC

**Fig. 4:**
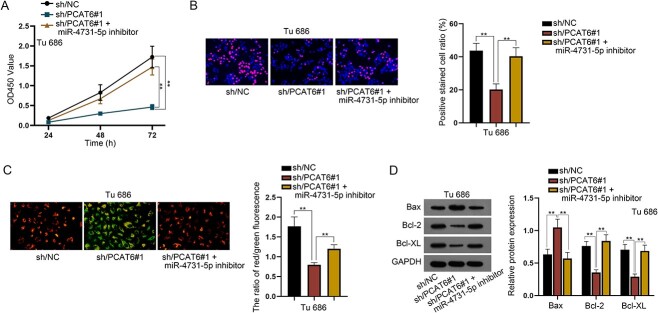
PCAT6/miR-4731-5p axis promoting the growth ability of LSCC. (**A**, **B**) CCK8 and EdU assays were conducted to test cell proliferation in sh/NC, sh/PCAT6#1 and sh/PCAT6#1 + miR-4731-5p inhibitor groups. (**C**) JC-1 assay was to assess cell apoptosis in different groups. (**D**) Western blot assay was to examine the protein levels of apoptosis-related factors in different groups. ^*^^*^P < 0.01.

To confirm PCAT6/miR-4731-5p axis in LSCC, rescue assays were implemented. After that, we divided experiment groups into sh/NC, sh/PCAT6#1 and sh/PCAT6#1 + miR-4731-5p inhibitor. Under this circumstance, CCK8 and EdU assays uncovered that cell viability was rescued by the miR-4731-5p inhibitor after cell proliferation was suppressed by PCAT6 silencing (Fig. 4A and B). On the contrary, PCAT6 reduction led to an increase in cell apoptosis which was completely offset by the miR-4731-5p inhibitor (Fig. 4C and D). The results clearly demonstrated that, following PCAT6 silencing, cell viability and proliferation were significantly impaired. Intriguingly, the introduction of the miR-4731-5p inhibitor effectively rescued these effects, highlighting the role of miR-4731-5p in counteracting the inhibitory impact of PCAT6 reduction. Collectively, PCAT6 sponged miR-4731-5p to exert its promoting role in LSCC.

## DISCUSSION

LSCC is a malignant tumor with high metastasis [13]. LncRNAs are regarded as novel biomarkers for LSCC due to their abnormal expression and modulation of malignant cell phenotypes [14]. LncRNA PCAT6 has been investigated in various kinds of human diseases [15]. For instance, as a ‘sponge’ for miR-513a-5p, lncRNA PCAT6 promotes the development of bladder cancer [16]. LncRNA PCAT6 influences chemoresistance in cervical cancer by targeting the miR-543/ZEB1 axis [17]. LncRNA PCAT6 contributes to the process of osteosarcoma via enhancing MDM2 expression [18]. Based on the series of functions and roles of PCAT6, we aim to investigate the role of PCAT6 in LSCC. As a result, PCAT6 was uncovered to be highly expressed in LSCC cells. The results of functional experiments indicated that PCAT6 promoted LSCC cell proliferation and restrained cell apoptosis.

The NOTCH signaling pathway plays a crucial role in tumorigenesis and cancer progression. NOTCH is a family of transmembrane receptors that are activated upon binding to ligands expressed on neighboring cells. In normal physiological conditions, the NOTCH pathway is involved in various cellular processes, such as cell fate determination, differentiation, proliferation and apoptosis. However, dysregulation of the NOTCH pathway has been implicated in the development and progression of various types of cancer [9]. Meanwhile, the involvement of lncRNAs in NOTCH signaling pathway regulates the occurrence and development of cancers [19]. As a family member of NOTCH family, NOTCH3 also mainly exerts oncogenic roles in cancers [20]. In our study, we found that the luciferase activity of NOTCH pathway was declined after PCAT6 was silenced. Moreover, we identified that PCAT6 could positively modulate NOTCH3 to activate NOTCH pathway. Furthermore, PCAT6 exacerbated cell proliferation and hindered cell apoptosis in LSCC via upregulating NOTCH3.

Naturally, we subsequently conducted experiments to figure out how PCAT6 mediated NOTCH3 expression in LSCC cells. Through subcellular fractionation assay, we observed that PCAT6 was chiefly distributed in the cytoplasm of LSCC cells, which signified us that PCAT6 might function at posttranscriptional level [21]. CeRNAs act as regulators by sequestering the target molecules and preventing them from interacting with their intended binding partners. This competition can impact various cellular processes, including gene expression, RNA stability and protein function [22]. Thereafter, we assumed that there was a shared miRNA binding to both PCAT6 and NOTCH3. MiR-4731-5p has been reported to act as tumor suppressors in glioma [23]. Furthermore, Ma *et al.* have verified that miR-4731-5p impedes cell migration, invasion, epithelial-mesenchymal transition (EMT) and stemness in nasopharyngeal carcinoma [24]. Through our investigation, the relationship among PCAT6, miR-4731-5p and NOTCH3 in LSCC cells was substantiated. Ultimately, rescue assays were performed to validate that PCAT6 aggravated the tumorigenesis of LSCC through sequestering miR-4731-5p.

Although we have conducted these studies on the role of PCAT6 in LSCC, they are far from sufficient and still have some limitations. For example, there is a lack of validation in clinical samples and evaluation of the future clinical application value, which requires further research. Additionally, it is unclear whether PCAT6 in LSCC functions solely as an endogenous competitor or if it may also interact with proteins to exert its effects. Our future research will focus on enriching clinical sample-related experimental validation of PCAT6 and further elucidating other mechanisms of action of PCAT6 in LSCC.

## CONCLUSIONS

In summary, we identified PCAT6 as a novel possible biomarker for LSCC and figured out its oncogenic role to promote cell proliferation and inhibit cell apoptosis. Besides, PCAT6 could target miR-4731-5p/NOTCH3 axis and modulate NOTCH pathway, which might provide a new therapeutic target for LSCC in the future.

## Supplementary Material

Figure_S1_rrae042

Figure_S2_rrae042

Figure_S3_rrae042

## Data Availability

Not applicable.
