# Supplementary figures and images for: LncRNA PCAT6 promotes the occurrence of laryngeal squamous cell carcinoma via modulation of the miR-4731-5p/NOTCH3 axis
Source: J Radiat Res. 2024 Jul 2;65(4):474–81. doi: 10.1093/jrr/rrae042 (PMC11262867; doi:10.1093/jrr/rrae042)

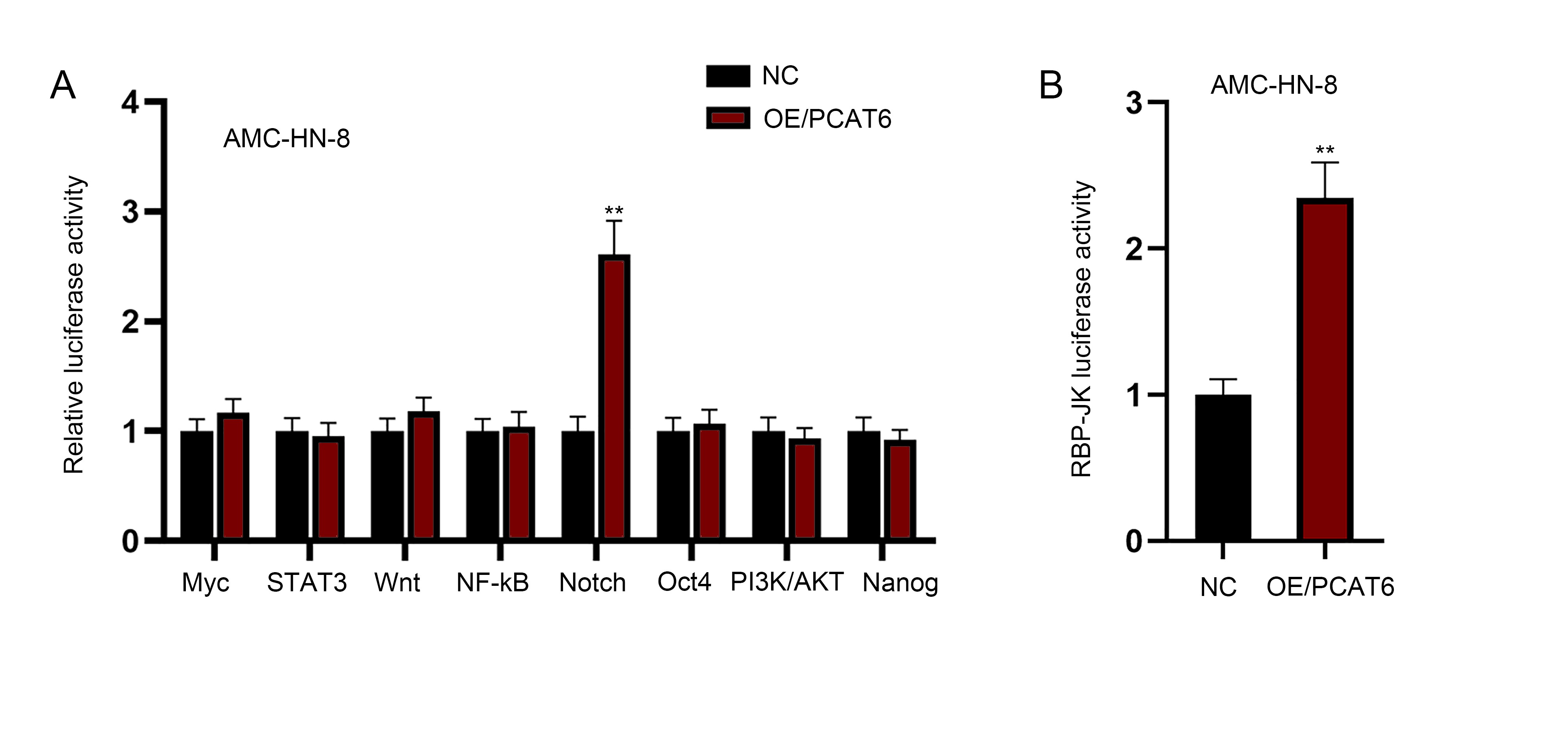

Supplement: Figure_S1_rrae042 [file figure_s1_rrae042.jpeg]

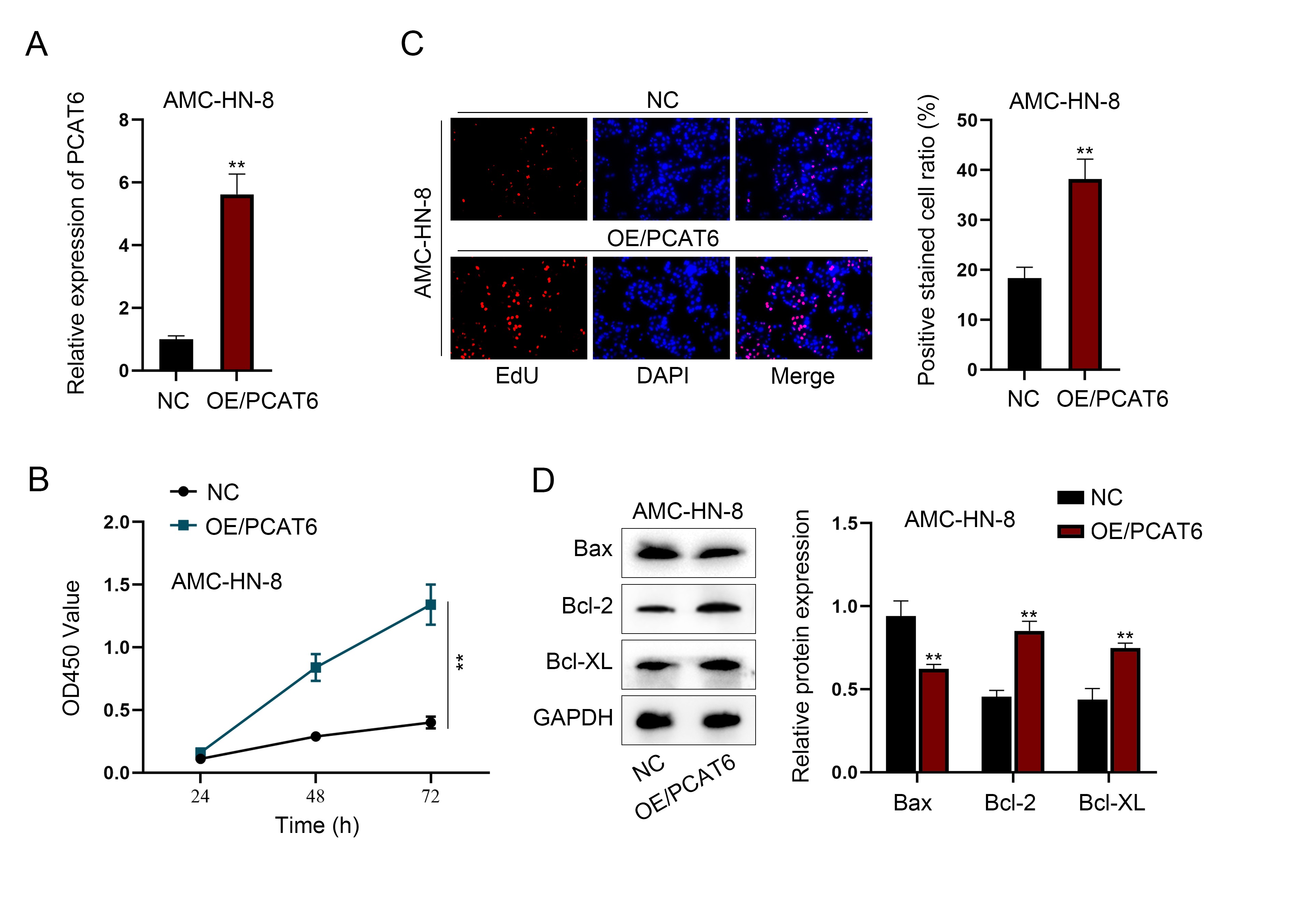

Supplement: Figure_S2_rrae042 [file figure_s2_rrae042.jpeg]

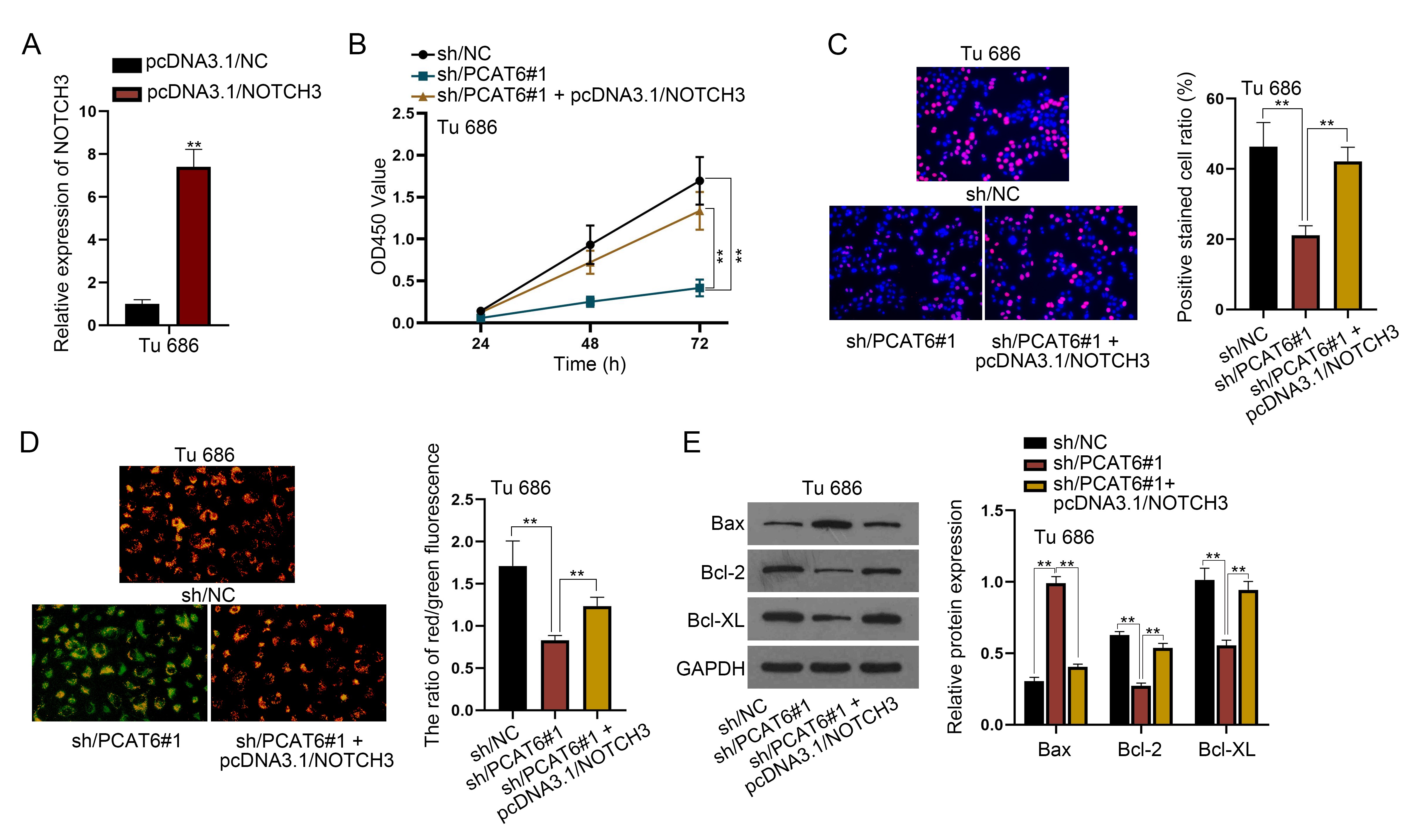

Supplement: Figure_S3_rrae042 [file figure_s3_rrae042.jpeg]
